# Carbon Derived from Pine Needles as a Na^+^‐Storage Electrode Material in Dual‐Ion Batteries

**DOI:** 10.1002/gch2.201700055

**Published:** 2017-08-29

**Authors:** Xiaohong Wang, Cheng Zheng, Li Qi, Hongyu Wang

**Affiliations:** ^1^ State Key Laboratory of Electroanalytical Chemistry Changchun Institute of Applied Chemistry Chinese Academy of Sciences Changchun 130022 China; ^2^ University of Chinese Academy of Sciences Beijing 100049 China; ^3^ School of Materials & Energy Guangdong University of Technology No. 100 Outer Ring West Road Guangzhou 510006 China

**Keywords:** carbon, dual‐ion batteries, graphite, pine needle

## Abstract

Pine needles are used as the precursor material to prepare hard carbon. Scanning electron microscopy, X‐ray diffraction, and N_2_ adsorption–desorption tests are carried out to characterize the surface, crystal, and pore structure of the material. The pine needle derived carbon (PNC) exhibits excellent Na‐ion storage ability. A dual‐ion battery of PNC/graphite using a Na^+^‐based organic electrolyte is constructed. The batteries display outstanding electrochemical performance: a superior energy density (200 Wh kg^−1^ at 131 W kg^−1^), high cut‐off voltage (4.7 V), and outstanding cycling stability (87.2% retention after 1000 cycles). In addition, the separate responses of the cathode and anode are investigated.

Recently, dual‐ion batteries (DIBs) have attracted increasing attention in the community of electric energy storage devices.^1^, ^2^, ^3^, ^4^, ^5^, ^6^ This kind of electric energy storage device is based on the storage of anions and cations at the positive and negative electrodes, respectively.^7^, ^8^, ^9^, ^10^, ^11^, ^12^ Therefore, one of their most noticeable advantages may come from the fact that graphite is commonly used as the positive electrode material. It is well known that graphite is economic, environmentally benign, and plentiful on the earth. Moreover, graphite can reversibly accommodate a considerable capacity of anions (over 100 mAh g^−1^) within a very high potential range (≈5 V vs Li/Li^+^, 4.7 V vs Na/Na^+^).^13^, ^14^, ^15^, ^16^, ^17^ The application of a positive graphite electrode can significantly elevate the working voltage and further enlarge the energy density of the total cell. By contrast, the selection of a suitable negative electrode in conjunction with a compatible electrolyte is an indispensable factor for the fulfillment of satisfactory DIBs. This issue has triggered many investigations exploring the storage of different cations with various negative electrodes.^18^, ^19^, ^20^, ^21^, ^22^, ^23^, ^24^ Among the large family of cations, lithium acts as the lightest charge carrier in nonaqueous electrolyte systems. However, lithium has to face its unfortunate fate of being a limited resource in the world. Hence, sodium has received increased attention as an alternative choice mainly because of its abundance and its similar physical and chemical properties to lithium.^25^, ^26^, ^27^ Therefore, the application of a Na^+^‐based organic electrolyte may be a valuable strategy for the development of DIBs in the future. It is noteworthy that the state‐of‐the‐art positive electrode materials based on sodium storage can deliver capacity values generally less than 150 mAh g^−1^ in potential ranges lower than 4 V versus Na/Na^+^.^28^, ^29^ Thus, the excellence of positive graphite electrodes will become more competitive in sodium‐based DIBs.

For DIBs, in contrast with the increased number of reports about anion intercalation within graphite,^30^, ^31^, ^32^ the investigations of anodes are insufficient. In theory, anode active materials with distinguished Na^+^ storage capacities in the low potential range can match the performance of positive graphite electrodes. Carbon materials should be a promising candidate for this task considering the safety of the devices they are utilized in, the availability of raw materials, and other factors.^33^ Lu and co‐workers have reported that soft carbon is a high‐performance anode material for sodium‐based DIBs.^34^ Based on the above, we predicted that hard carbon and graphite will also become an appropriate couple. Nevertheless, researchers are plagued by the high production cost and complex synthesis procedures for synthesizing hard carbon.^35^ For the sake of tackling this issue, employing biomass resources to synthesize carbon materials is an available and convenient strategy.^36^, ^37^, ^38^, ^39^, ^40^ Biomass materials can exhibit multilevel, multidimensional, and hierarchical porous structures, which can be reserved in the derived carbons and are favorable for the penetration of electrolytes and ion diffusion.^41^ During synthesis, they can be used as their own templates, which solves many problems (e.g., the complicated processes, long periods, expensive raw materials, and pollution involved using other materials) caused by adopting artificial templates.^42^, ^43^, ^44^ In addition, natural biomaterials contain heteroatoms, such as nitrogen, boron, and phosphorous, that can provide extra active sites for Na^+^ storage. In this paper, we synthesized hard carbon by simply calcining pine needles without activation (Figure S1, Supporting information). Dried pine needles contain crude protein, fat, fiber, and various sugars (glucose, fructose, galactose, and sucrose),^45^ which are all carbon sources. Moreover, pine needles are slender. The average leaf length of conifer is 2.3 cm with 75% of leaves shorter than 6 cm. The radius of the conduits that transport the sugar and water in the phloem cells is below 3 μm.^46^ Therefore, the vessels in the pine needle carbon (PNC) may help to construct pores that are accessible for Na^+^ storage and transportation. Moreover, pine needles are so soft that the breaking machines used in the kitchen can grind them into a powder, which reduces fabricating costs.

**Figure** **1**a–d shows the scanning electron microscopy (SEM) images of the pine needle powder, PNC4, PNC, and graphite, respectively. The particles of the graphite (KS6) demonstrate a plate‐like shape, and their sizes are quite small (generally ≤6 μm).^13^ By contrast, the pine needle powder, PNC4 and PNC have larger particle sizes than the graphite, especially PNC, with much bigger sizes over 20 μm. On the other hand, their surface morphologies are also very different from that of the graphite. Pine oil is a component that should be taken into account. The boiling point of pine oil is 153–175 °C. During the carbonization of the pine needle powder at 400 °C, the pine oil and carbon were separated. The volatilization of the pine oil is beneficial for forming porous carbon. Many cavities and wrinkles are observed on the surface, which may have originated from the texture of the pine needles. Furthermore, the surface of the PNC appears rougher, and the particle size is larger. It seems that after treatments with HCl and NaOH to remove the impurities, the surface of the carbon becomes etched. In addition, the adjacent PNC4 particles join together to form PNC powder with greater sizes after being baked at 1000 °C. In Figure 1d, the X‐ray diffraction (XRD) pattern of PNC4 mainly shows a broad diffraction peak at 24.5° (2θ), which indicates the presence of amorphous carbon. A few impurity peaks are observed in the XRD pattern of PNC4. By contrast, for PNC, these impurity peaks almost disappear which evidences the effective removal of many impurities after the HCl and NaOH treatments. The XRD pattern of the PNC exhibits a few graphite‐like layers developed in the short range. The specific surface area was calculated using the Brunauer–Emmett–Teller method. The specific surface areas of the PNC and graphite are 12.6 m^2^ g^−1^ (Table S1, Supporting Information) and 14.5 m^2^ g^−1^,^13^ respectively, which are far lower than that of active carbon (AC). The low specific surface area values of the active materials (both PNC and graphite) indicate that the contribution of the electric double‐layer capacitance to the energy storage is negligible.

**Figure 1:**
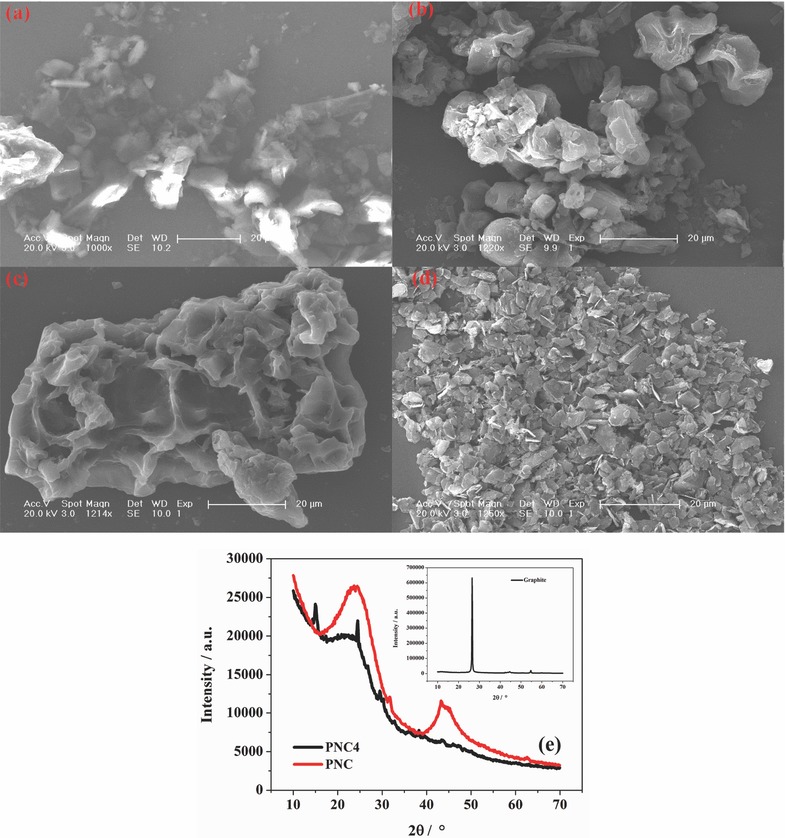
SEM images of the a) pine needle power, b) PNC4, c) PNC, and d) graphite. e) XRD patterns of PNC4, PNC, and graphite (insert).

The PNC electrode demonstrates an outstanding Na^+^ storage capacity in the low potential region and a stable structure during the electrochemical reactions (Figure S2, Supporting Information). The diffusion of Na^+^ within the crystalline framework of PNC occurs easily (Figure S3 and Table S2, Supporting Information). The active anode material is a primary factor that influences the performance of the DIB. Based on the abovementioned results, PNC satisfies the requirement of DIBs.

We assembled DIBs with a presodiated PNC anode and a graphite cathode. The working mechanism (**Figure** **2**) of the PNC/graphite battery is distinct from the traditional “rocking‐chair” batteries. During the charging process, PF_6_^−^ anions are intercalated into the graphite positive electrode, and the Na^+^ cations are intercalated into the PNC negative electrode at the same time. When a DIB discharges, both the PF_6_^−^ anions and Na^+^ cations leave the graphite and PNC electrodes, respectively. Typical charge–discharge curves of the DIB with a current density of 500 mA g^−1^, a voltage of 0.8–4.7 V, and a mass ratio of 3 are displayed in **Figure** **3**a. It is noted that there is a distinct charge plateau at ≈4.5 V and a discharge plateau at ≈4.0 V, which are caused by both the graphite and PNC electrodes. Figure 3b presents the d*Q*/d*V* differential curve in which each peak corresponds to each platform of the charge/discharge curve. The PNC/graphite DIB delivers a specific capacity of 155 mAh g^−1^ and 146 mAh g^−1^ during the first charge–discharge step with a very high initial Coulombic efficiency (CE) of 93.8%. The high capacity and efficiency at such a high current density (500 mA g^−1^) are higher than those of most sodium‐ion batteries reported previously. The Na^+^ is predoped in the PNC electrode to adjust the potential to a value at which the anode could work properly because the intercalation and deintercalation of the Na^+^ ions are not completely reversible. The rest of the Na^+^ ions left inside of the PNC electrode causes a lower potential in the negative electrode, which contributes to the high working voltage producing high capacity. The process of predoping Na^+^ ions into the PNC electrode improves the performance of DIBs remarkably (Figure S4, Supporting Information). In addition, the high initial CE is attributed to the process of doping Na^+^ into the PNC anode prior to assembling the DIB. The initial capacity loss is always caused by the formation of the solid electrolyte interphase (SEI) film. The SEI is generated while predoping Na^+^ into the PNC electrode (Figure S5a,b, Supporting Information). As a consequence, the initial irreversible capacity of the DIB with the presodiated PNC electrode is insignificant. The DIB exhibits an excellent long cycling performance, 87.2% retention of the capacity after 1000 cycles (Figure 3c). The shapes of the curves remain unchanged, and there are still obvious platforms in the 1000th cycle. We observe that the structures of the graphite and PNC are not damaged (Figure S5c, Supporting Information). The attenuation of capacity is due to the slow decomposition of the electrolyte. The Ragone plot of the PNC/graphite DIB is shown in Figure 3d. A high energy density of 200 Wh kg^−1^ is achieved at a power density of 131 W kg^−1^ and still remains at 43.8 Wh kg^−1^ with a power density of 1716 W kg^−1^. These values are comparable to those of commercial lithium‐ion batteries. Figure 3e presents the typical charge–discharge curves of the PNC/graphite DIB at various current density values. With the increase in the current density, slight changes in voltage plateau occur, suggesting slight polarization in the DIB. The rate performance of the DIB is further displayed in Figure 3f. The discharge capacities are 155, 147, 141, 130, and 98 mAh g^−1^ at the current density of 50, 100, 200, 500, and 1000 mA g^−1^ with a Coulombic efficiency of 92.4%, 95.4%, 97.1%, 97.6%, and 98.1%, respectively. When the current density returns to 50 mA g^−1^, the discharge capacity returns to 155 mAh g^−1^, revealing the good stability (Figure S5d, Supporting Information).

**Figure 2:**
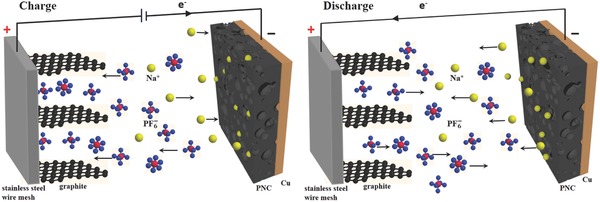
Schematic illustration of the working mechanism of the PNC/graphite DIB.

**Figure 3:**
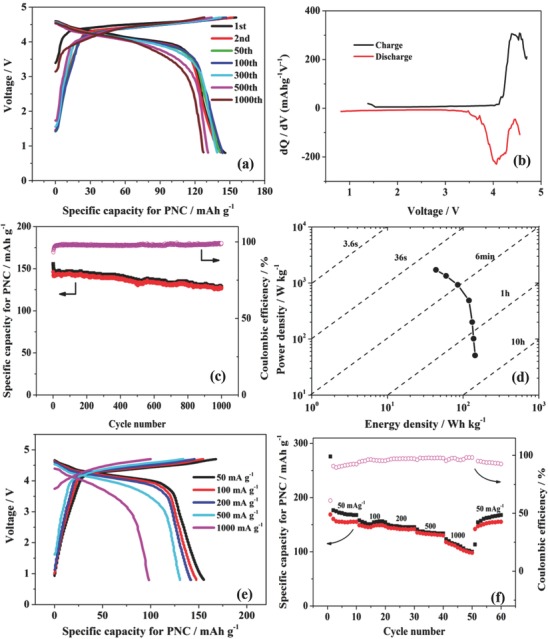
a) Charge–discharge curves. b) d*Q*/d*V* differential curves. c) Long cycling performance with a current density of 500 mA g^−1^ and cut‐off voltage range of 0.8–4.7 V. d) Ragone plot of the PNC/graphite DIB. e) Charge–discharge curves at the tenth cycle for each current density and f) the corresponding capacities and Coulombic efficiencies of the PNC/graphite DIB at different current densities. The mass ratio of graphite/PNC is 3.

To further analyze the respective electrochemical behaviors of both the graphite cathode and PNC anode precisely, the separate potentials were measured against a heavy AC reference electrode (**Figure** **4**a).^47^ Here, we consider the charge process as an example. Within the battery voltage range of 0.8–4.0 V, the potentials of the graphite and PNC electrodes change abruptly, which indicates that the electrochemical reactions barely proceed. Then, lengthy plateaus near 1.7 and −2.7 V versus AC are in the potential profile of the graphite and PNC electrode, which are characteristic of PF_6_^−^ anions and Na^+^ cations intercalation, respectively. Meanwhile, the changing trends of the bottom and top potentials of the graphite cathode and PNC anode with the increasing cycle number are exactly the same (Figure 4b,c). The potentials rise slowly and are maintained at a stable value. The potential of the graphite positive electrode ranges from 1.25 to 1.97 V versus AC, and the potential of the PNC negative electrode changes from −2.72 to 0.46 V versus AC with the DIB cycling between 0.8 and 4.7 V. The stabilized potential ensures the excellent long cycling performance of DIBs.

**Figure 4:**
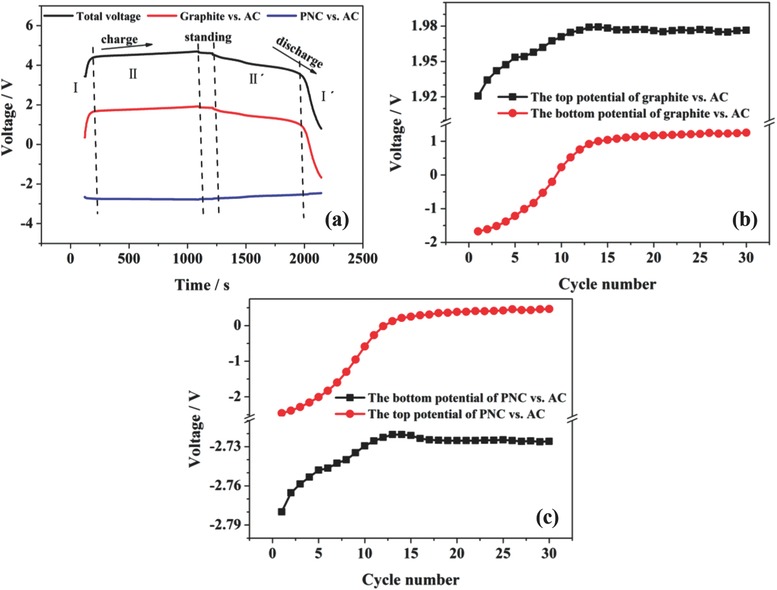
a) Potential profiles of the positive graphite electrode and negative PNC electrode versus the AC quasi‐reference electrode in the PNC/graphite DIB. The relationship between the cycle number and the bottom potential and top potential of the b) graphite cathode and c) PNC anode versus the AC electrode.

Carbon has been synthesized used pine needles as a raw material through a simple pyrolysis method and applied as the negative electrode in DIBs. The PNC/graphite DIB using the Na^+^‐based organic electrolyte can reach a high cut‐off voltage (4.7 V), which is higher than the working potential of most sodium‐ion batteries. Moreover, the energy density is 200 Wh kg^−1^ at 131 W kg^−1^, which is comparable with that of lithium‐ion batteries. The impressive performance of the DIB benefits from the nice match between the anode and cathode and the excellent properties of the PNC. The PNC anode in the DIB can deliver a capacity of 140 mAh g^−1^ at a high current of 500 mA g^−1^. After 1000 cycles, the capacity remains at 127 mAh g^−1^. In addition, the PNC anode exhibits a good rate performance, 155 mAh g^−1^ at 50 mA g^−1^ and 98 mAh g^−1^ at 1000 mA g^−1^. Furthermore, the individual potential actions of the cathode and anode during DIBs working have been investigated.

## Experimental Section

*Preparation of the PNC*: First, pine needles (collected from fallen leaves in October) were ground to powder after being washed and dried with an ordinary pulverizer used in the kitchen. Then, the powder was calcined at 400 °C for 4 h under nitrogen flow. The obtained product was marked as PNC4. Next, the PNC4 was boiled with 3 M HCl (aq) for 1 h and washed with deionized water until it reached pH ≈7 in order to remove the metals. Afterward, the PNC4 was boiled with 2 M NaOH (aq) for 1 h and washed to remove the silicon elements. Finally, the sample was calcined at 1000 °C for 3 h under nitrogen flow. The final product was labeled as PNC.

*Characterization of the PNC and Graphite*: SEM images were performed by Philips XL 30. XRD patterns were obtained using a Bruker D8 advance diffractometer with Cu Kα radiation (λ = 1.5418 Å). N_2_ adsorption/desorption isotherms were measured at 77 K with the Micromeritics ASAP 2020 analyzer.

*Electrochemical Measurements*: To prepare the positive electrodes, graphite KS6 was mixed (Timcal Co. Ltd., Switzerland) with teonized acetylene black, and then, the mixture was pressed on a stainless steel wire mesh. The PNC electrodes were prepared by mixing PNC, super‐p and polyvinylidene fluoride in a mass ratio of 80:10:10 employing *N*‐methyl pyrrolidinone as a solvent to form a homogeneous slurry. Then, the slurry was coated onto a copper foil. After being dried at 80 °C, the foil was punched into a 14 mm diameter electrode. The electrolyte was 1 M NaPF_6_ dissolved in a mixture of ethylene carbonate and ethyl methyl carbonate (1:2 by volume). All the coin cells were assembled in a glove box (Mikrouna Co. Ltd.) filled with an argon atmosphere. Before assembling the DIB, sodium ions were predoped into the PNC electrode. The Na/PNC half‐cells were discharged from open circuit voltage to 0 V versus Na/Na^+^ at a low current density to finish the predoping process. Cyclic voltammetric tests were performed on CHI660D electrochemical workstation. Galvanostatic charge–discharge tests and rate capacity tests of the PNC/graphite coin cell were conducted using a Land 2001A cell test system (Wuhan, China).

## Conflict of Interest

The authors declare no conflict of interest.

## Supporting information

Supplementary
