# Supplementary material for: Carbon Derived from Pine Needles as a Na+‐Storage Electrode Material in Dual‐Ion Batteries
Source: Glob Chall. 2017 Aug 29;1(7):1700055. doi: 10.1002/gch2.201700055 (PMC6607337; doi:10.1002/gch2.201700055)
Supplement: Supplementary file 1 — Supplementary [file GCH2-1-1700055-s001.pdf]

Copyright WILEY-VCH Verlag GmbH & Co. KGaA, 69469 Weinheim, Germany, 2017.

Global Challenges Open Access

Supporting Information

for *Global Challenges*, DOI: 10.1002/gch2.201700055

Carbon Derived from Pine Needles as a Na^+-Storage Electrode Material in Dual-Ion Batteries

*Xiaohong Wang, Cheng Zheng, Li Qi, and Hongyu Wang**

Copyright WILEY-VCH Verlag GmbH & Co. KGaA, 69469 Weinheim, Germany, 2016.

Supporting Information

Title (Carbon derived from pine needles as a Na^+-storage electrode material in dual-ion batteries)

*Xiaohong Wang, Cheng Zheng, Li Qi, and Hongyu Wang**

Figure S1. (Illustration of the synthesis of the carbon derived from pine needles)

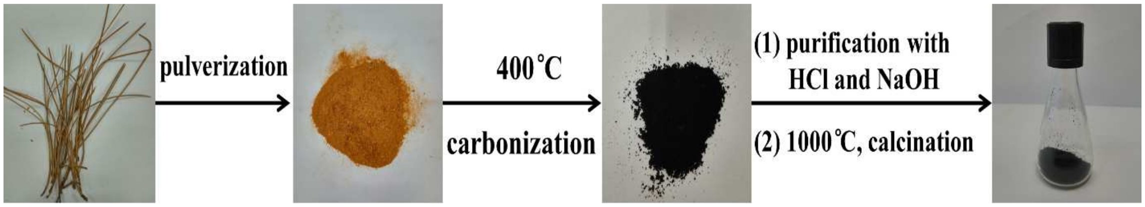

Figure S2. ((a) The charge-discharge curves and (b) cycling stability curves of the Na/PNC cell between 2.0 and 0 V with a current density of 50 mA g^{-1})

(a)

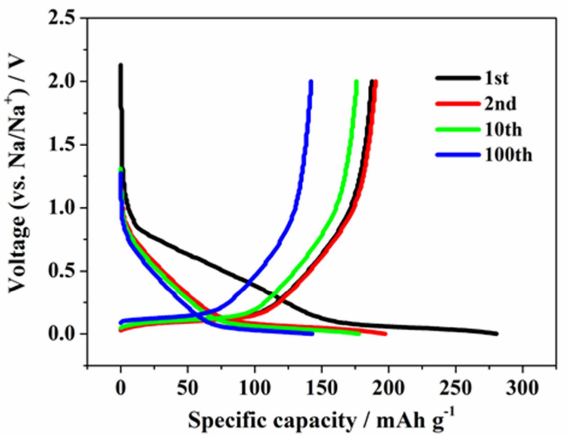

(b)

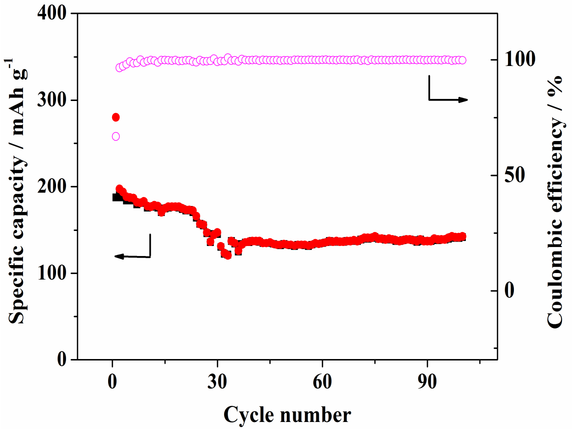

Figure S3. ((a) Cyclic voltammograms of the Na/PNC half-cell at different scan rates in the voltage range of 0-2.5 V and (b) the relationship between i_p and $v^{1/2}$. The apparent diffusion rate of Na^+ (D_{Na^+}) can be calculated using i_p as a function of $v^{1/2}$ through the Randles-Sevcik equation.)

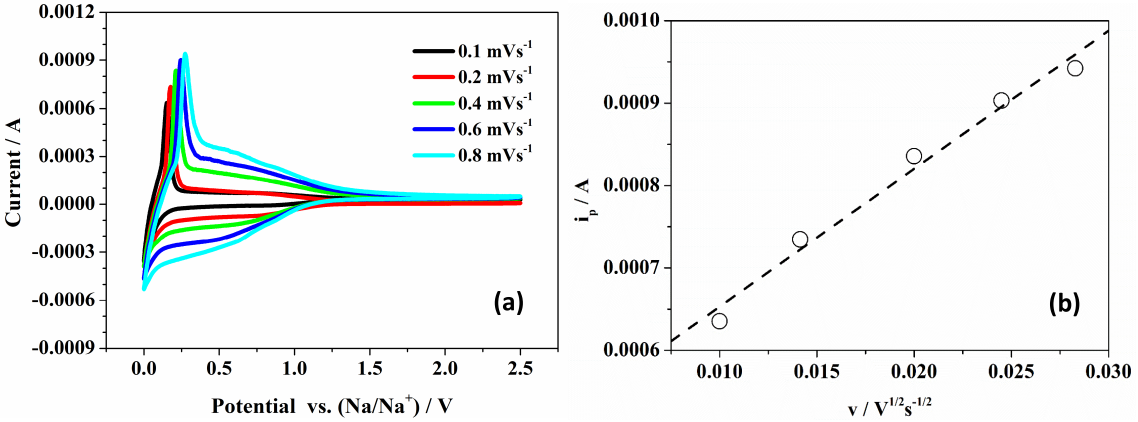

Figure S4. (Long cycling performance of the DIBs with pre-sodiated and pristine PNC anodes with a current density of 500 mA g^{-1} and cut-off voltage range of 0.8-4.7 V. The graphite/PNC mass ratio is 1.)

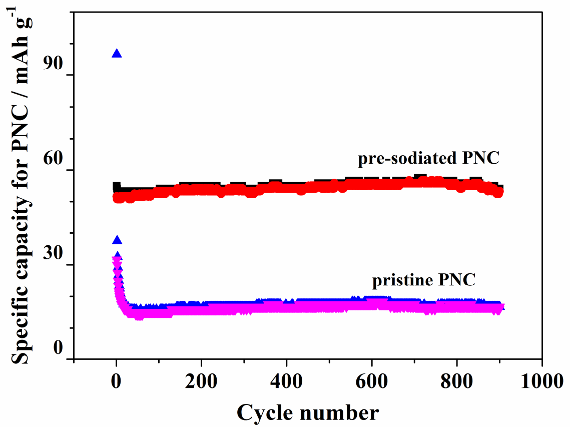

Figure S5. SEM images of a (a) pristine PNC anode, (b) pre-sodiated PNC anode, (c) PNC anode after 100 cycles, and (d) PNC anode after cycles with various current densities.

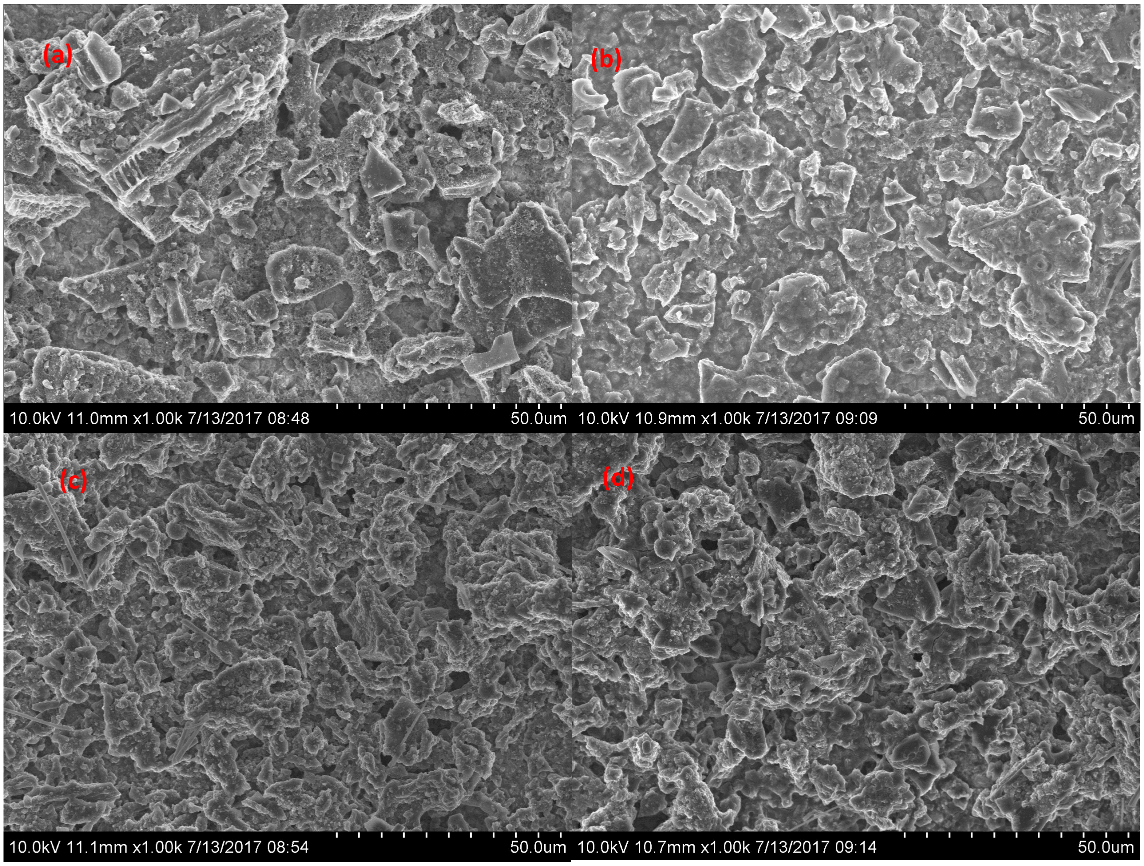

Table S1. (The BET specific surface area, pore volume average pore diameter and pore size distribution based on nitrogen adsorption)

	PNC
Specific surface area (m^2 g^{-1})	12.60
Pore volume (cm^3 g^{-1})	0.016
Average pore diameter (nm)	2.84

Table S2. (Diffusion coefficient of Li^+ (or Na^+) obtained using CV)

Samples	electrolyte	D_{Li^+}	D_{Na^+}	Reference
porous microspheres of $LiNi_{0.6}Co_{0.2}Mn_{0.2}O_2$	1 M $LiPF_6$ / EC+EMC (1:1 in vol%)	10^{-8}	---	[1]
$Li[Li_{0.2}Ni_{0.13}Co_{0.13}Mn_{0.54}]_{1-0.03}Mo_{0.03}O_2$	1 M $LiPF_6$ / EC+DMC (1:1 in vol%)	4.61×10^{-9}	---	[2]
$LiFePO_4$	1 M $LiPF_6$ / PC	4.06×10^{-11}	---	[3]
$LiFePO_4$	0.5 M Li_2SO_4 / H_2O	2.05×10^{-10}	---	[3]
LiFePO4 / polypyrrole	1 M $LiPF_6$ / EC+EMC (3:7 in vol%)	10^{-14}		[4]
Nano-Si	1 M $LiPF_6$ / EC+DMC (1:1 by wt%)	5.1×10^{-12}	---	[5]
Si film	1 M $LiPF_6$ / EC+EMC+DMC (1:1:1)	10^{-11}	---	[6]
C_{60}-coated Si	1 M $LiPF_6$ / EC+EMC+DMC (1:1:1)	10^{-9}	---	[6]
Commercial $Li_4Ti_5O_{12}$	1 M $LiPF_6$ / EC+DMC (1:1 in vol%)	3.47×10^{-12}	---	[7]
hierarchical mesoporous nest-like $Li_4Ti_5O_{12}$	1 M $LiPF_6$ / EC+DMC (1:1 in vol%)	2.58×10^{-11}	---	[7]
Carbon-coated $Li_4Ti_5O_{12}$	1 M $LiPF_6$ / EC+EMC+DMC (1:1:1 in vol.%)	6.42×10^{-11}	---	[8]
Surface-modified $Li_4Ti_5O_{12}$	1 M $LiPF_6$ / EC+DMC+EMC (1:1:1 in vol.%)	1.834×10^{-11}	---	[9]
$Li_3V_2(PO_4)_3$	1 M $LiPF_6$ / EC+DMC (1:1 by wt%)	10^{-10}	---	[10]
$LiSn_2(PO_4)_3$	1 M $LiPF_6$ / EC+DMC+DEC (1:1:1 in vol%)	9.02×10^{-13}	---	[11]
Carbon fiber	1 M $LiClO_4$ / PC	10^{-10}	---	[12]
$Na_7V_4(P_2O_7)_4(PO_4)/C$	1 M Na_2SO_4 / H_2O	---	3.9×10^{-11}	[13]
$Na_3V_2(PO_4)_3$	1 M $NaPF_6$ / PC	---	10^{-13}	[14]
$Na_3V_2(PO_4)_3$	1 M $NaClO_4$ / PC	---	3.63×10^{-10}	[15]
$K_3V_2(PO_4)_3$	1 M $NaClO_4$ / EC+DEC (1:1 by wt%)	---	4.0×10^{-9}	[16]
$K_3V_2(PO_4)_3/C$	1 M $NaClO_4$ / EC+DEC (1:1 by wt%)	---	1.6×10^{-8}	[16]
$Na_3Fe_2(PO_4)_3$	1 M $NaClO_4$ / EC+DMC (1:1 by vol%)	---	2.4×10^{-8}	[17]
P2-$Na_{0.66}[Li_{0.22}Ti_{0.78}]O_2$	1 M $NaPF_6$ / EC+DEC (4:6 in vol%)	---	$1\ \times10^{-10}$	[18]
a-SnO_2/GA	1 M $NaClO_4$ / EC +PC (2:1 in vol%) with 10 vol% FEC	---	1.958×10^{-16}	[19]
PNC	1 M $NaPF_6$ / EC+EMC (1:2 in vol%)	---	1.63×10^{-9}	This work

References

[1] Z. Zheng, X. Guo, S. Chou, W. Hua, H. Liu, S. X. Dou, X. Yang, *Electrochim. Acta* 2016, 191, 401.

[2] X. Jin, Q. Xu, X. Liu, X. Yuan, H. Liu, *Ionics* 2016, 22, 1369.

[3] P. He, X. Zhang, Y. Wang, L. Cheng, Y. Xia, *J. Electrochem. Soc.* 2008, 155, A144.

[4] X. Wang, H. Yoshitake, Y. Masaki, H. Wang, *Electrochim. Acta* 2014, 130, 532.

[5] N. Ding, J. Xu, Y. X. Yao, G. Wegner, X. Fang, C. H. Chen, I. Lieberwirth, *Solid State Ionics* 2009, 180, 222.

[6] A. A. Arie, J. K. Lee, *Phys. Scr.* 2010, T139, 014013.

[7] J. Chen, L. Yang, S. Fang, S. Hirano, K. Tachibana, *J. Power Sources* 2012, 200, 59.

[8] J. B. Kim, D. J. Kim, K. Y. Chung, D. Byun, B. W. Cho, *Phys. Scr.* 2010, T139, 014026.

[9] B. Yan, M. Li, X. Li, Z. Bai, J. Yang, D. Xiong, D. Li, *J. Mater. Chem. A* 2015, 3, 11773.

[10] X. H. Rui, N. Yesibolati, S. R. Li, C. C. Yuan, C. H. Chen, *Solid State Ionics* 2011, 187, 51.

[11] W. Cui, J. Yi, L. Chen, C. Wang, Y. Xia, *J. Power Sources* 2012, 217, 77.

[12] M. W. Verbrugge, B. J. Koch, *J. Electrochem. Soc.* 1996, 143, 600.

[13] C. Deng, S. Zhang, Y. Wu, *Nanoscale* 2015, 7, 487.

[14] N. Böckenfeld, A. Balducci, *J. Solid State Electrochem.* 2014, 18, 959.

[15] H. Wang, D. Jiang, Y. Zhang, G. Li, X. Lan, H. Zhong, Z. Zhang, Y. Jiang, *Electrochim. Acta* 2015, 155, 23.

[16] X. Wang, C. Niu, J. Meng, P. Hu, X. Xu, X. Wei, L. Zhou, K. Zhao, W. Luo, M. Yan, L. Mai, *Adv. Energy Mater.* 2015, 5, 1500716.

[17] Y. Liu, Y. Zhou, J. Zhang, Y. Xia, T. Chen, S. Zhang, *ACS Sustainable Chem. Eng.* 2017, 5, 1306.

[18] Y. Wang, X. Yu, S. Xu, J. Bai, R. Xiao, Y. Hu, H. Li, X. Yang, L. Chen, X. Huang, *Nat. Commun.* 2013, 4, 2365.

[19] L. Fan, X. Li, B. Yan, J. Feng, D. Xiong, D. Li, L. Gu, Y. Wen, S. Lawes, X. Sun, *Adv. Energy Mater.* 2016, 6, 1502057.
